# Intrafraction motion of the prostate during an IMRT session: a fiducial-based 3D measurement with Cone-beam CT

**DOI:** 10.1186/1748-717X-3-37

**Published:** 2008-11-05

**Authors:** Judit Boda-Heggemann, Frederick Marc Köhler, Hansjörg Wertz, Michael Ehmann, Brigitte Hermann, Nadja Riesenacker, Beate Küpper, Frank Lohr, Frederik Wenz

**Affiliations:** 1 Department of Radiation Oncology, University Medical Center Mannheim, Theodor-Kutzer-Ufer 1-3, Mannheim, Germany

## Abstract

**Background:**

Image-guidance systems allow accurate interfractional repositioning of IMRT treatments, however, these may require up to 15 minutes. Therefore intrafraction motion might have an impact on treatment precision. 3D geometric data regarding intrafraction prostate motion are rare; we therefore assessed its magnitude with pre- and post-treatment fiducial-based imaging with cone-beam-CT (CBCT).

**Methods:**

39 IMRT fractions in 5 prostate cancer patients after ^125^I-seed implantation were evaluated. Patient position was corrected based on the ^125^I-seeds after pre-treatment CBCT. Immediately after treatment delivery, a second CBCT was performed. Differences in bone- and fiducial position were measured by seed-based grey-value matching.

**Results:**

Fraction time was 13.6 ± 1.6 minutes. Median overall displacement vector length of ^125^I-seeds was 3 mm (M = 3 mm, Σ = 0.9 mm, σ = 1.7 mm; M: group systematic error, Σ: SD of systematic error, σ: SD of random error). Median displacement vector of bony structures was 1.84 mm (M = 2.9 mm, Σ = 1 mm, σ = 3.2 mm). Median displacement vector length of the prostate relative to bony structures was 1.9 mm (M = 3 mm, Σ = 1.3 mm, σ = 2.6 mm).

**Conclusion:**

a) Overall displacement vector length during an IMRT session is < 3 mm.

b) Positioning devices reducing intrafraction bony displacements can further reduce overall intrafraction motion.

c) Intrafraction prostate motion relative to bony structures is < 2 mm and may be further reduced by institutional protocols and reduction of IMRT duration.

## Background

Accurate interfractional patient repositioning before prostate radiotherapy has become possible in the era of Image-Guided RadioTherapy (IGRT, [1]) with several in-room systems such as stereotactic ultrasound [2,3], beacon responders [4] and kV/MV cone- or fan-beam CT based methods [5-7]. Correction of interfractional movement improves radiation treatment accuracy if geometrical changes are within certain limits [8,9].

However, the prostate has to be considered an inter- and intrafractionally moving target due to permanent changes in bladder and rectum filling [10]. Motion of the prostate has been analysed e.g. by MR imaging. Padhani *et al*. have shown by cine MRI measurements a significant displacement in the antero-posterior direction [11]. Ghilezan *et al*. have shown a correlation between rectal filling and intrafraction motion [12]. Similar data were provided by Mah *et al*. and Nichol *et al*. [13,14].

Intensity Modulated RadioTherapy (IMRT) allows dose escalation with creation of steep dose gradients [15]. Since a step-and-shoot IMRT treatment fraction requires up to 15 minutes [16-18], intrafraction motion due to changing bowel and bladder filling might be an issue [19]. Intrafraction motion should have an impact on PTV margins [20,21] and dose distribution [22,23] and several approaches are currently pursued for real time target tracking for compensation [24]. Such approaches allow measuring intrafraction motion and include sonographic tracking [25], tracking based on implanted fiducials [26,27] or beacon responders [4,28] and even linac-mounted MRI [29].

Comprehensive 3D volumetric data are, however, scarce [12,30-32] and data published so far may overestimate prostate mobility if an appropriate preparatory protocol is used [33].

We therefore set out to broaden the available database for patients undergoing an institutional preparatory protocol designed to reduce inter- and possibly intrafraction prostate motion comparing pre- and post-treatment on-board 3D imaging based on fiducials with a gantry-mounted cone-beam CT (CBCT; Elekta Synergy^®^).

## Methods and patients

### Patients, treatment planning and delivery

78 CBCTs of 39 fractions in 5 patients with intermediate risk prostate cancer [34,35] were evaluated. Patients were treated with a 6 MV linear accelerator (Elekta Synergy^®^, Elekta Inc., Crawley, U.K.) with step-and-shoot IMRT plans (Corvus^®^, NAS/Nomos, Cranberry Township, USA; 45 Gy) as part of a combined protocol after ^125^I-seeds-implantation. Stranded ^125^I-seeds (Rapid Strand, Oncura, Castrop-Rauxel, Germany) were used with a diameter of 0.5 mm and a length of 3 mm. Median number of ^125^I-seeds was 50.

Treatment planning CT datasets were acquired and processed by each patient using an institutional policy (moderately full bladder, empty rectum using an enema before planning CT) as published previously [2,36]. Patients were also instructed to empty their rectum and bladder and drink 500 ml water before each treatment fraction. The procedure was explained and informed consent was obtained. Typical IMRT plans consisted of 7 or 9 incident beams.

### Cone-beam volume imaging and patient repositioning

Before delivering a treatment fraction, kV CBCT volume imaging was performed. Approximately 610 projection images were acquired during a 360°-rotation using the presets of Elekta (for a single projection: 120 kV, 25 mA, 40 ms). Projections were processed to 3D volume images by the XVI^® ^(X-Ray Volume Imaging) software of Elekta using "high resolution" [36]. Patient position was corrected on-line based on matching the ^125^I-seeds [36] in the CBCT to the planning CT images with a small alignment clip box considering the prostate (and the ^125^I-seeds) only. Matching and translational position correction was performed by an automatic grey value algorithm included in the XVI software and always controlled visually by a physician. The XVI algorithm has been extensively tested by phantom-experiments regarding accuracy in case of matching based on ~50 fiducials also in case of physiologic soft tissue deformations [36,37]. Rotational errors were not corrected. Bone position was recorded by offline matching regarding the bony structures with an alignment clip box considering the whole bony pelvis [36]. Immediately after IMRT delivery, a second CBCT imaging was performed.

### Offline image analysis and data processing

The second CBCT was evaluated offline as follows: Changes in bone position (patient intrafraction movement, considering the whole pelvis; alignment clip box including the whole bony pelvis) and fiducial position (overall fiducial/prostate displacement, alignment clip box considering only the ^125^I-seeds/prostate) were measured by offline grey-value matching ensuring user-independence. Prostate motion relatively to the pelvic bone structures was calculated on a patient-to-patient basis.

Overall mean value (mv) and overall standard deviation (SD) and median value of all translational displacements in each direction were calculated for the matching results. Group systematic error (M), standard deviation of the systematic error (Σ) and standard deviation of the random error (σ) were calculated [38,39]. All data were evaluated on a patient-by-patient basis.

The length of the translational displacement vector was calculated with the following formula: v = √(x^2 ^+ y^2 ^+ z^2^).

## Results

### Duration of a step-and-shoot IMRT fraction

Time between the pre- and post-treatment CBCTs was 13.6 ± 1.6 minutes (mv ± SD).

### Overall intrafraction prostate displacement

Median overall displacement vector length of ^125^I-seeds was 3 mm (M = 3 mm, Σ = 0.9 mm, σ = 1.7 mm; M: group systematic error, Σ: SD of systematic error, σ: SD of random error).

mv ± SD of overall displacement of ^125^I-seeds was 0.4 ± 2 mm, 1.1 ± 3.9 mm and 1.3 ± 4.5 mm in × (left-right), y (cranio-caudal) and z (antero-posterior; AP) directions (mean length of overall displacement vector 5.1 ± 3.9 mm).

### Intrafraction displacement of bony structures

Median displacement vector of bony structures was 1.84 mm (M = 2.9 mm, Σ = 1 mm, σ = 3.2 mm).

mv ± SD of displacement of bony structures was 0.2 ± 2.0 mm, -0.3 ± 1.6 mm and -0.2 ± 4.3 mm in x, y and z directions (mean length of overall displacement vector 3.1 ± 3.9 mm).

### Intrafraction displacement of prostate relative to pelvic bones

**Figure 1:**
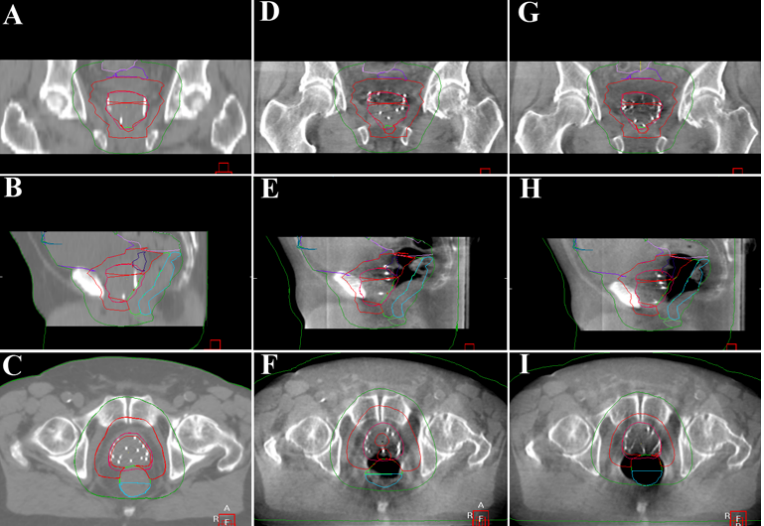
**Planning CT (A-C) and CBCT images of a patient immediately before (D-F) and after (G-I) an IMRT fraction**. Prostate position differs due to differences in rectal filling during therapy caused by rectal gas not present on treatment planning (in this case -7.4 mm in AP, 1.1 mm in left-right and -1.0 mm in cranio-caudal directions). Planning CT was acquired due to the intitutional policy using enema. This patient marks the maximum unidirectional displacement observed. In general patients with larger differences in rectal filling between treatment planning CT and daily online cone beam CT are asked to empty their rectum and drink water before another treatment attempt.

Median intrafraction motion of the prostate due to changes in bladder/bowel filling (relative to bony structures) was 1.9 mm (displacement vector length; M = 3 mm, Σ = 1.3 mm, σ = 2.6 mm).

mv ± SD of intrafraction motion of the prostate due to changes in bladder/bowel filling relative to bone structures is -0.2 ± 3.2 mm, -1.5 ± 3.7 mm and -1.5 ± 4.8 mm in x, y and z directions (mean length of overall displacement vector 5.4 ± 4.8 mm). A patient example with a relative large difference in pre- and post-treatment soft tissue position due to a moderately enlarged rectum due to gas that shifted during therapy is shown on Fig. 1. The enlargement in rectal volume seen in this patient was at the limit of what is tolerated at our department. Patients with a larger difference in rectal filling between treatment planning CT are taken off the treatment table and instructed to defecate/pass gas before treatment.

**Figure 2:**
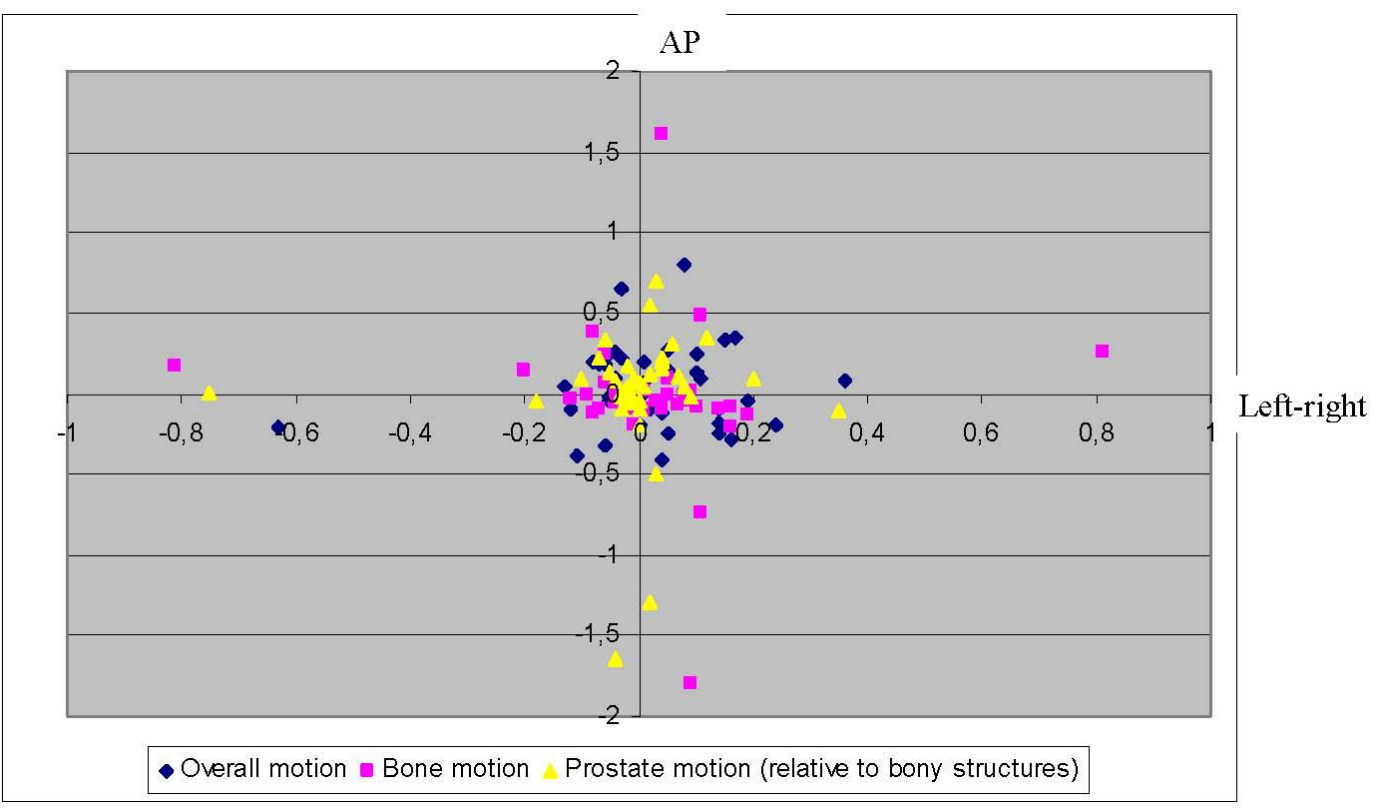
**Overall intrafraction motion vs bony displacement only vs soft tissue displacement**. Overall intrafraction motion (blue); bony displacement only (pink) and displacement of the sof tissue structures relative to the bony anatomy (yellow) on a patient-to-patient basis in × (left-right; abscissa) and z (AP; ordinate) directions.

All results are summarised in Table 1 and displacements on a patient-to-patient basis are shown on Fig 2.

**Table 1:** Analysis of prostate intrafraction motion

**n = 39**		**ΔTranslation (mm)**
**Overall motion**	**Median vector length**	3
	
	**M**	3
	
	**Σ**	0.9
	
	**σ**	1.7

**Motion of bony anatomy**	**Median vector length**	1.8
	
	**M**	2.9
	
	**Σ**	1
	
	**σ**	3.2

**Soft tissue motion (relative to pelvic bones)**	**Median vector length**	1.9
	
	**M**	3
	
	**Σ**	1.3
	
	**σ**	2.6

M: group systematic error, Σ: standard deviation of the systematic error; σ: standard deviation of the random error.

## Discussion

Modern IGRT technologies allow precise repositioning, target-volume-visualization and even daily re-planning [40-42] before treatment delivery so that interfraction motion of various target sites in the body can be considered a solved problem [43,44]. Patient- and organ motion, however, also has an intrafractional component which has to be considered, too. In some anatomic sites (lung- and liver tumors, upper abdominal targets), intrafraction motion can be extreme due to e.g. respiratory or cardiac motion if not addressed properly. In case of the prostate, continuous changes in bladder and bowel filling may cause a significant intrafraction motion component that is currently still accounted for by PTV margins and dose distribution. Based on measurements with the Calypso™ system, the prostate may, in some cases, move as much as > 3–5 mm already in the first 5–10 minutes of the treatment [28], however with a significant individual variation among patients [10,28]. The percentage of patients with these extreme movements, however, is small (3D-offset exceeding 5 mm was observed in 15% of 1157 fractions in 35 observed patients, [28]) in a patient cohort that apparently underwent no specific preparatory protocol. Other groups have also published data without specifying if a preparatory protocol was used. Using 2D kV projections and 3 implanted fiducials, Letourneau *et al*. have shown a small intrafraction motion with a SD of 0.9 mm [31]. Wu *et al*. calculated the effect of intrafraction motion on dose distribution and showed that intrafraction motion worsens target coverage, its effect of course being larger for small margins than for larger margins [32]. MRI based data already suggest that intrafraction motion is smaller in comparison to interfraction motion, with an expected displacement of the prostate of < 3 mm for 20 minutes in case of patients with an empty rectum [12,30]. However, this was not the case in patiens with a full rectum [12,30], which underlines the importance of using institutional protocols before planning CT and each therapy fraction. We therefore set out to further evaluate this parameter in the actual treatment setting in exactly such a patient cohort that was appropriately prepared before treatment planning CT and each treatment fraction.

In this work, we analysed 3D geometric data about intrafraction motion of the prostate with pre- and post-treatment on-board imaging based on fiducials with a gantry-mounted cone-beam CT for the first time. The novelty of our data lie in the 3D online volumetric assessment of inrafraction prostate motion. We have shown that during a single step-and-shoot IMRT fraction of about 15 minutes, the overall motion of the prostate is relatively small (median displacement vector length 3 mm with a group systematic error of 3 mm). While we could not assess spurious, reversible movements of the prostate during beam-on-time, this seems to be a less relevant event based on on-line beacon data [10,28]. To analyse the deformational component of the motion, fiducial to fiducial mapping would be very interesting, however it is not possible with the current vendor-provided XVI™ matching algorithm and was therefore not addressed because it would have comprised a large extent of manual matching. Qualitative evaluation, however, showed that the deformational component was minor in comparison to translation and tilt and the evaluation of the matching algorithm showed, that minor deformations do not affect evaluation of translation and tilt [37]. However, actually deformable matching algoritms which are able to perform a fiducial-to-fiducial based matching are being developed in our department and are planned to be subject of further work. The intrafraction motion of the prostate consists of two components: motion of the bony structures caused by e.g. relaxation of the patient, coughing, non-compliance etc., and motion of the soft tissue structures relative to the bone due to e.g. changing bowel or bladder filling. The bony motion component can be minimized by patient education and improved immobilisation techniques with devices such as stereotactic body frames [45], leg holder immobilisation devices [46] or customized body pillows formed by vacuum suction [47]. The soft tissue motion relative to bony structures due to changing bowel and bladder filling can be reduced by institutional policies such as training of the patients to have an empty rectum and a relatively full bladder before each radiation session as suggested by other groups and used for our patients [33]. If pre-delivery image guidance shows that, e.g., the rectum is filled (as opposed to the planning CT), patients can be repositioned after emptying the rectum.

The extent of prostate motion is correlated to treatment delivery time [11]. Further reduction of IMRT delivery time to ~3 minutes with novel methods such as IMAT (Intensity Modulated Arc Therapy)/VMAT (Volumetric Modulated Arc Therapy) will reduce overall target motion [48-50]. Given the small magnitude of intrafractional motion and the prospect for further dramatic reduction of treatment times in the near future, the impact of this component on the compound positioning error will likely lose relative importance.

Intrafraction motion has, in addition to the translational displacement, which was analysed in this report, also rotational and deformational components [51]. Intrafraction rotation [36,52] could possibly be compensated (if necessary) in the future with real time target tracking and treatment tables with 6 degrees of freedom (HexaPOD™). In this work, we did not analyse the interfraction motion of each individual fiducial, however, studies about inter- and intrafraction prostate deformation are currently being performed with a self-developed deformable matching algorithm at our department. While the translational intrafractional motion is small in our series and is accounted for with PTV margins of 5 mm, an additional deformational component may well be negligible. Faster treatment paradigms such as IMAT/VMAT may aid further in the reduction of PTV margins.

## Conclusion

a) The median length of the displacement vector of the prostate during a ~13 min IMRT session is < 3 mm. This amount of intrafraction motion has to be considered in choosing PTV margins even in patient cohorts that undergo daily online image guidance.

b) Positioning devices reducing intrafraction bony displacements can further reduce overall intrafraction prostate motion.

c) Intrafraction motion of the prostate represented by ^125^I-seeds relative to bony structures is < 2 mm in patients appropriately prepared according to institutional protocols (full bladder, empty rectum). It may be further reduced by reduction of IMRT duration (e.g. by IMAT; Intensity Modulated Arc Therapy, ~ fraction time 3 minutes).

## Abbreviations

CBCT: Cone-beam CT; IMRT: Intensity Modulated RadioTherapy; IGRT: Image-Guided RadioTherapy; XVI: X-ray Volume Imaging; kV: kilovoltage; MV: megavoltage; PTV: Planning Target Volume; EPID: Electronic Portal Imaging Device; EBRT: External Beam Radiation Therapy; 3D: 3-dimensional; IMAT: Intensity Modulated Arc Therapy; VMAT: Volumetric Modulated Arc Therapy; mv: mean value; SD: standard deviation.

## Competing interests

This work was partially supported by grants from Elekta GmbH, Hamburg, Germany.

## Authors' contributions

JBH conceived the study, drafted the manuscript the supervised data acquisition. NR and BK acquired data, FMK evaluated the CBCTs offline. HW, ME and BH acquired data and were involved in data analysis. FL and FW supervised the project and finalized the manuscript together with JBH.
